# Using the theory of planned behavior and self-identity to explore women’s decision-making and intention to switch from combined oral contraceptive pill (COC) to long-acting reversible contraceptive (LARC)

**DOI:** 10.1186/s12905-019-0772-8

**Published:** 2019-06-20

**Authors:** Andrea L. DeMaria, Beth Sundstrom, Amy A. Faria, Grace Moxley Saxon, Jaziel Ramos-Ortiz

**Affiliations:** 1 College of Health and Human Sciences, Purdue University, West Lafayette, IN USA; 2 Department of Communication, College of Charleston, Charleston, SC USA; 3 Emory University School of Medicine, Atlanta, GA USA; 4 Department of Consumer Science, Purdue University, West Lafayette, IN USA

**Keywords:** Theory of planned behavior; LARC, Self-identity, SEM, IUD, Implant

## Abstract

**Background:**

***\***Most college women use the combined oral contraceptive pill (COC) despite more effective long-acting reversible contraceptive (LARC) methods (e.g., IUDs and implant) being available. Resistance to change methods may be impacted by how a woman identifies with being a COC-user.

**Methods:**

Data were collected via 186 web-based surveys distributed to female students attending a university in the southeastern United States (Mean age = 20.0 ± 1.; range = 18–22). Structural equation modeling (SEM) determined TPB fit in understanding LARC intention.

**Results:**

SEM results received acceptable fit (χ2 (670, *N* = 186) *p* < 0.01, Comparative Fit Index (CFI) of 0.84, and Normative Fit Index (NFI) of 0.75). A Root Mean Square Error of Approximation (RMSEA) of 0.09 was produced, with a 90% confidence interval of 0.08 to 0.09. Including self-identity in the model yielded similar fit, with χ2 (866, *N* = 186) *p* < 0.01, CFI of 0.83, and NFI of 0.73. Self-identity and attitude pathways were significant (*p* < 0.01) toward intention, extending the TPB model.

**Conclusions:**

The TPB proved to be acceptable in understanding COC users’ intention to obtain LARC. Results provide direction for LARC messaging tailored toward COC users and self-identity.

**Electronic supplementary material:**

The online version of this article (10.1186/s12905-019-0772-8) contains supplementary material, which is available to authorized users.

## Background

Unintended pregnancy rates in the United States remain high, despite recent declines [1, 2]. Nearly half of all pregnancies in the United States are unintended, with higher rates among at-risk groups, such as racial minorities, adolescent and college-aged women [3]. Among these groups, challenges of low socioeconomic status, increased risk of reproductive issues, and high rates of induced abortions are most common, which may result in poor health (e.g., chronic disease, cervical tear, mental health issues) and economic outcomes for women [1, 2]. Unplanned births are often attributed to issues in contraception misuse, nonuse, or use of ineffective methods [4]. The Food and Drug Administration (FDA), a federal agency part of the United States Department of Health and Human Services, has approved several highly effective contraceptive methods [5]. However, the combined oral contraceptive pill (COC), although repeatedly associated with high user-error and poor reliability [6, 7], remains the most commonly used method. Long-acting reversible contraceptive (LARC) methods, including intrauterine devices (IUDs) and the implant, are preferable for reducing unintended pregnancy among reproductive-aged women [1, 6, 8, 9] due to their effectiveness and ability to decrease user error [10]. Contrary to positive satisfaction rates and attitudes [4, 6, 11], LARC uptake as a primary contraceptive method remains minimal [7, 8, 12]. Researchers identified this resistance to change to a LARC method, regardless of its favorability among current COC users, as the *paradox of inertia* [6]*.* The paradox of inertia identifies the phenomenon of acknowledging LARC as a superior choice and perceiving significant disadvantages of the COC yet resisting to switch to a LARC method.

Sundstrom et al. [13] conducted qualitative research analyzing existing LARC campaigns and media messaging. Despite exposure to existing informative and supporting messages, the majority of college-aged women were unaware of LARC’s greater efficacies [13]. Additional barriers to LARC uptake included: 1) perceptions of IUDs and implants as ‘foreign objects,’ causing hesitation of insertion; 2) resistance to change from a current method perceived as more ‘normal’; and 3) perceived lack of physician knowledge about the LARC method [4, 14, 15]. Pritt et al. [4] identified cost and lack of insurance coverage awareness as additional concerns. Misconstrued beliefs (e.g., potential pain) [16], underestimated effectiveness, parental influence on contraceptive choice, and patient-perception of physicians’ lack of knowledge and training in LARC counseling, have also been identified as limitations [4, 17]. Analogous to these findings, Berlan et al. [18] studied physicians’ attitudes toward LARC counseling and found most female pediatricians shared negative attitudes toward IUDs, often due to outdated information. Physicians expressed insufficient knowledge about the methods and saw them as more suitable for older women than adolescents [18]. These beliefs often transferred to personal ideologies, preventing pediatricians’ support, recommendation, and discussion of LARCs as viable options for their patients [18]. With the FDA’s 2016 approval of new LARC methods [1], understanding barriers, misconceptions, and attitudes toward uptake among physicians and patients has become increasingly important.

Attitude alone, however, may be limiting in its ability to predict health behavior; thus, exploring additional factors influencing contraceptive choice and intention to use LARC may increase uptake and contribute to a decrease in unintended pregnancies. Ajzen’s theory of planned behavior (TPB) [19] postulates an individual’s future behavior can be predicted by intention to perform the behavior. Intention is conceptualized through one’s attitude toward the behavior, subjective norm, and perceived behavioral control over performing the behavior [19]. The TPB has been used to explain diverse health behaviors (e.g., tobacco use, breast examination and condom uptake) [20–22], demonstrating its validity in predicting health behavior intention. The TPB has also been used to understand women’s intention for LARC uptake [8]. DeMaria et al. [8] utilized structural equation modeling (SEM) to test the theory and found attitude and subjective norm significantly predicted intention of LARC uptake among reproductive-aged women; however, perceived behavioral control did not show significant results, contrary to other health behavior research [20–22]. Researchers suggested this finding was consistent with low LARC uptake rates seen in the United States, and recommended developing educational messages targeting attitude, norms, and increasing knowledge about IUDs and implants [8]. This was exemplified among women who had been using any prescription contraceptive and felt that being informed might empower them to make the change [8]. Though menstruation is suggested to be linked to self-identity [23], little is known about self-identity’s role in contraceptive choice and the TPB. Research grounded in this theory may help explain why women who are aware of the benefits of LARC methods still resist changing.

Self-identity is defined as characteristics that help construct an internal self-perception, often focusing on one’s multiple life roles [20, 24]. Behaviors are internalized and contribute to an individual’s self-understanding. These internalizations can guide decisions and often influence health behaviors, which has been seen as a viable extension of the TPB [19–21, 24–27]. Furthermore, understanding self-identity’s direct influence on behavioral intention has proven to elicit improved model fit statistics when compared to indirect influences [23]. Høie et al. studied motivation to quit smoking and found self-identity, as well as the TPB factors, were significant predictors of smoking intention among undergraduate students.

Fekadu and Kraft [28] found self-identity, attitude, and subjective norm were significant predictors of future condom use among adolescents. Past behavior moderated the effect between self-identity and intention, showing high habitual contraceptive practices decreased self-identity’s ability to predict intention. An individual’s contraceptive habits become reflexive and are more influential on intention than the other factors —an effect which may help explain women’s lack of LARC uptake despite being aware of its benefits and effectiveness [28]. Further confirming these findings, Zerbini et al. [29] showed self-identity can predict intention to purchase generic pharmaceuticals among an Italian sample. Using SEM, researchers found purchasing a generic drug was an important part of an individual’s self-concept, as it was the greatest influencer of intention. Similarly, Pierro et al. [26] utilized SEM to assess the validity of the TPB, including self-identity to predict intention to participate in leisure activities. Attitude, perceived behavioral control, and self-identity pathway coefficients were significant predictors of intentions.

Despite some health behavior-related examples, little is known regarding the impact of self-identity on contraceptive choice, particularly that of LARC methods. Health decisions are often the result of ambivalent attitudes [30], as they are diverse in context, environmental factors, and exogenous complexities. Understanding the TPB validity and the role of self-identity in predicting LARC uptake behaviors warrants investigation. Examining these relationships in women currently using COCs who are aware of LARC methods (i.e., IUDs and implant) may lead to a greater understanding of the paradox of inertia and resistance to change. We seek to explore if the TPB is a viable lens through which to examine and understand COC users’ intention to obtain LARC.

## Methods

College-aged women attending an urban, liberal arts institution in the southeastern United States participated in the study by completing an online questionnaire (see Additional file 1: LARC Questionnaire). Participants were primarily recruited via e-mail and social media advertisements. Upon survey completion, all participants were invited to enter their name into a draw for a chance to win a gift basket worth $30 (1 in 30 odds of winning). Of the 482 completed surveys, 186 participants were current COC user who were aware of IUDs and/or implant and were included in the final analyses.

### Measures

Data were collected in Fall 2016, with items pertaining to: demographics, the TPB constructs, and self-identity.

#### Demographics

To further conceptualize the study sample, age, sexual orientation, and race were included among survey items.

#### Theory of planned behavior

A complete listing of all TPB questionnaire items is located in Table 1. TPB factors were measured and adapted from Ajzen’s seven-point, bipolar Likert scales [31], traditionally used in similar studies. An expanded attitude construct containing 12 items was developed to fully capture the impact other perceptions have on attitude toward LARC. Items to capture perceived benefits for adopting the behavior were included, as this can also influence attitude [32].

**Table 1:** Descriptive statistics for TPB & self-identity constructs^a^

Questionnaire Items	Mean	SD
Attitude		
1. To me, obtaining an IUD or Implant seems		
*Extremely Difficult/Extremely Easy*	4.41	1.75
*Extremely Frightening/Extremely Comforting*	3.55	1.73
*Extremely Painful/Extremely Painless*	3.18	1.54
2. Choosing an IUD/Implant as my primary birth control method would be		
*Extremely Harmful/Extremely Beneficial*	5.06	1.62
*Extremely Inconvenient/Extremely Convenient*	5.24	1.68
*Extremely Irresponsible/Extremely Responsible*	5.48	1.67
*Extremely Unhealthy/Extremely Healthy*	4.83	1.71
3. Using an IUD or Implant would be more convenient for my lifestyle than other contraceptive methods, such as the pill.	4.91	1.94
4. IUDs and Implants are more effective at preventing pregnancy than other contraceptive methods, such as the pill.	5.15	1.69
5. An IUD or Implant would be able to control my acne as well as other contraceptive methods, such as the pill.	3.98	1.44
6. An IUD or Implant would be as effective at reducing menstrual cramps as other contraceptive methods, such as the pill.	4.13	1.60
7. An IUD or Implant would be as effective at regulating my menstrual cycle as other contraceptive methods, such as the pill.	4.67	1.63
Subjective Norm		
8. The IUD and Implant are good contraceptive options for women in my peer group.	5.80	1.52
9. My friends would support my decision to use an IUD or Implant as my primary contraceptive method.	6.13	1.40
10. My mother or female mentor would support my decision to use an IUD or Implant as my primary contraceptive method.	5.26	1.92
11. My sexual partner would support my decision to use an IUD or Implant as my primary contraceptive method.	6.79	1.77
12. If I used an IUD or Implant as my primary contraceptive method, I would be able to relate to my peers.	4.51	1.74
Perceived Behavioral Control		
13. I am confident in my ability to obtain an IUD or Implant if I desired to use it as my primary contraceptive method.	5.66	1.68
14. Obtaining an IUD/Implant as my primary contraceptive method would be		
*Not at all up to me/Completely up to me*	6.30	1.30
*Not at all under my control/Completely under my control*	6.11	1.43
15. My health care provider would support my decision to obtain an IUD or Implant.	5.82	1.38
16. My health insurance would pay for an IUD or Implant.	4.69	1.68
17. I know where to go if I wished to obtain an IUD or Implant as my primary contraceptive method.	5.81	1.69
Intention		
18. I intend to research more about the IUD or Implant in the future.	4.63	1.89
19. I intend to research if my insurance plan covers the IUD or Implant.	4.49	1.96
20. I intend to research facilities in my area that offer IUD or Implant insertions.	4.02	1.96
21. I intend to discuss the IUD or Implant as an option for my primary form of contraception with my health care provider at my next appointment.	3.77	2.04
22. I intend to discuss the IUD or Implant as an option for my primary form of contraception with my health care provider in the future.	4.24	2.04
23. I intend to learn more about the process of getting an IUD or Implant and how I may go about obtaining one in the future.	4.41	2.01
24. I intend to talk to my friends about the IUD or Implant in the future.	4.25	1.95
25. I intend to talk to my mom or female mentor about the IUD or Implant in the future.	4.01	2.01
26. I intend to talk to my partner about the IUD or Implant in the future.	3.99	2.06
27. I intend to ask IUD and Implant users about their experiences with the methods to determine if an IUD or Implant might be right for me.	4.77	1.92
28. I can see myself using an IUD or Implant as my primary contraceptive method in the next 12 months.	3.20	2.05
29. I can see myself using an IUD or Implant as my primary contraceptive method in the future.	4.37	2.13
30. I intend to take the steps to obtain an IUD or Implant as my primary contraceptive method in the future.	3.81	2.05
31. I intend to change my contraceptive method from the pill to the IUD or Implant.	3.38	2.07
Self-Identity		
32. Taking the pill every day is an important part of who I am.	4.44	1.96
33. I am the type of woman who would use an IUD or Implant as my primary birth control method.	4.45	1.91
34. I see myself as responsible enough to take the pill every day; the IUD and Implant are not for women like me. (reverse coded)	4.13	1.90
35. Having a monthly period is an important part of my identity as a woman.	3.09	2.15
36. Taking the pill every day protects my future fertility better than other contraceptive methods, like the IUD or Implant.	3.39	1.68

^a^Each item ranged between 1 = strongly disagree/extremely harmful/etc. to 7 = strongly agree/extremely beneficial/etc

Subjective norm and perceived behavioral control were also measured, each using five-item scales. LARC Intention was measured using 16 seven-point bipolar adjective scales consisting of multiple LARC scenarios and potential behaviors. Higher values on each of the scales indicate higher levels of accordance with each of the items measured. For example, a value of seven in response to the question “IUDs and Implants are more effective at preventing pregnancy than other contraceptive methods, such as the pill” would indicate a more favorable attitude surrounding the question asked.

#### Self-identity

Items assessing self-identity were adapted from previous studies in relation to contraceptive use [28]. An example item included: ‘Taking the pill every day is an important part of who I am’ (1 = strongly disagree; 7 = strongly agree). Three additional items were added to further conceptualize self-identity, and account for additional aspects found to influence a woman’s self-concept (e.g., fertility and ability to menstruate) [6]. This provided insight into self-identity and its ability to explain LARC intention, addressing this literature gap. Intention is expected to be lower as self-identity as a pill user increases.

### Data analyses

Quantitative data analysis was used to assess significance level of the TPB factors and relationships among them. This approach allows for increased generalizability of findings in comparison to qualitative methods often found within existing literature on LARC attitudes, perceptions, and intentions. SEM was used to understand TPB model validity in predicting LARC intention and the extension of the model to include self-identity.

Each item was operationally defined according to the item factor loadings and error terms; items that did not load onto the factors were not used in the final survey of this study. Outliers and missing data were previously removed. Initial descriptive statistics were assessed with IBM-SPSS 22.0, which was also used to conduct SEM and assess the model’s validity in predicting LARC intention. The model ran primarily with the latent variables associated with the TPB (attitude, subjective norm, and perceived behavioral control) to assess model fit. Cronbach’s alpha was used as an indicator of model reliability. A second model to test self-identity as an exogenous variable was examined and differences in model fit were identified. Confirmatory factor analysis was conducted by using SEM as a method to understand whether paths were significant and relevant fit of the model.

## Results

### Demographics

Mean age of the 186 participants was 20.0 ± 1.1 years (range = 18–22). Most participants (20.40%, *n* = 38) indicated they had utilized a COC for 3.2 ± 2.1 years. The majority of participants (91.4%, *n* = 170) self-identified as heterosexual, while a small portion self-identified as bisexual (5.9%, *n* = 11) or asexual (0.5%, n = 1). Participants self-identified as white or Caucasian (90.0%, *n =* 168), black or African American (5.4%, *n =* 10), or Hispanic or Latino (5.0%, *n* = 9).

### Theory of planned behavior constructs

All item scores are presented as Mean ± Standard Deviation in Table 1. Item averages range from 1 (negative) to 7 (positive); the closer the mean is to 1 or 7, the stronger the relationship with that adjective. Means above a 4 indicate a more positive outcome. Obtaining an IUD/implant was found to be easy (4.4 ± 1.8) versus frightening (3.6 ± 1.7) or painful (3.2 ± 1.5). Choosing the IUD/implant was seen as beneficial (5.1 ± 1.2), convenient (5.2 ± 1.1.7), and healthy (4.8 ± 1.7). Greater support for IUD/implant use would come from a sexual partner (6.8 ± 1.8) over a female mentor, mother, or friends. Intention to *ask* current IUD/implant users about their experiences with LARC methods prior to choosing it as their primary method (4.8 ± 1.9) was the highest rated intention item. In addition, intention to *use* an IUD/implant as a primary contraceptive method was greater in the future (4.4 ± 2.1) (one with an unspecified timeframe) than intention to use within a 12-month timeframe (3.2 ± 2.1). Some women saw themselves as the type of person who would use an IUD/implant (4.5 ± 1.9), while others felt responsible enough to take the COC every day and thought IUD/implants were not for women like them (4.1 ± 1.9). Having a monthly period was also an important part of a woman’s identity (3.1 ± 2.2) with significance (0.2, *p* < 0.05).

### Structural equation modeling

**Fig. 1:**
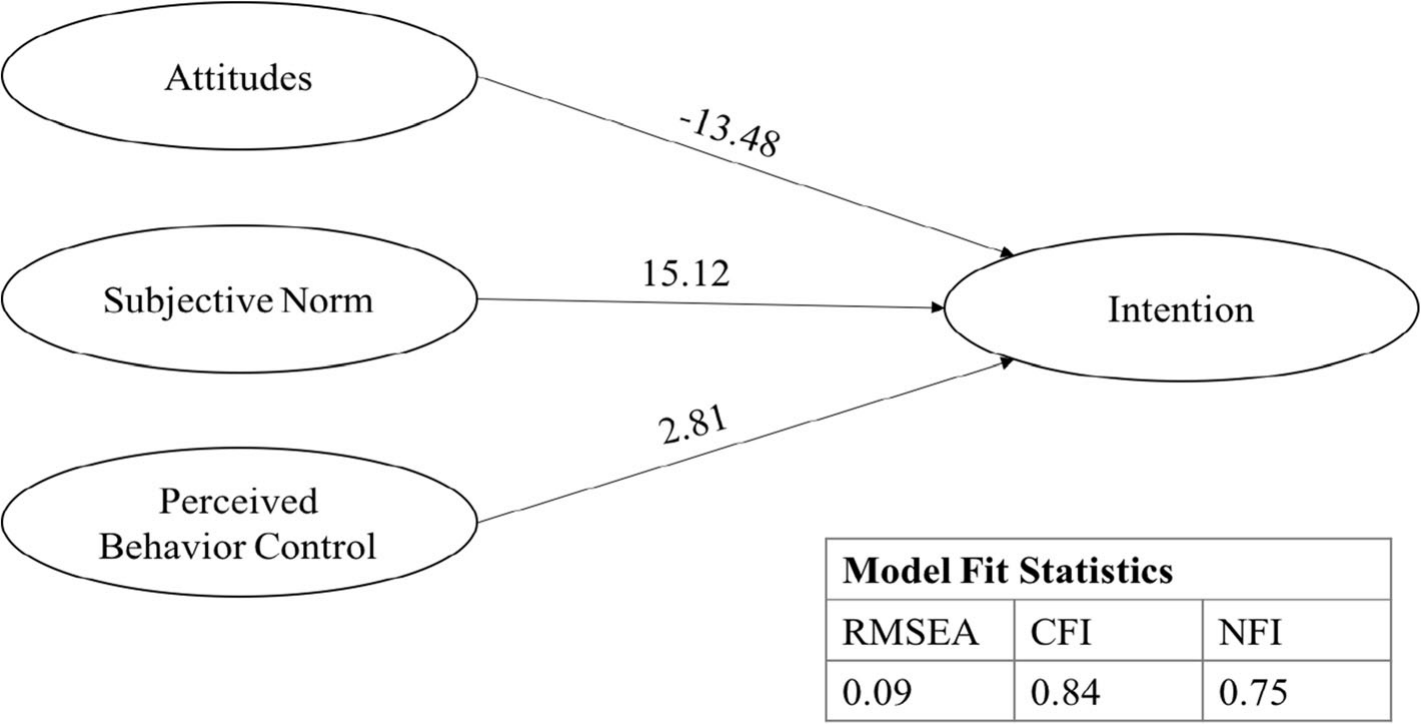
TPB model. Initial SEM results without latent factor of self-identity. Note: Independent, latent variable covariance paths not shown for simplicity

SEM was used to initially test TPB validity in measuring LARC intention (Fig. 1). Model fit statistics indicated χ2 (670, *N* = 186) *p* < 0.01, Comparative Fit Index (CFI) of 0.84, and Normative Fit Index (NFI) of 0.75. The model produced a Root Mean Square Error of Approximation (RMSEA) of 0.09 with a 90% confidence interval of 0.08 to 0.09. Standardized regression weight for perceived behavioral control, subjective norm, and attitude resulted in 2.8, 15.1, and − 13.5 respectively, with no significance. The TPB model alone was shown to be acceptable fit in understanding LARC intention.

**Fig. 2:**
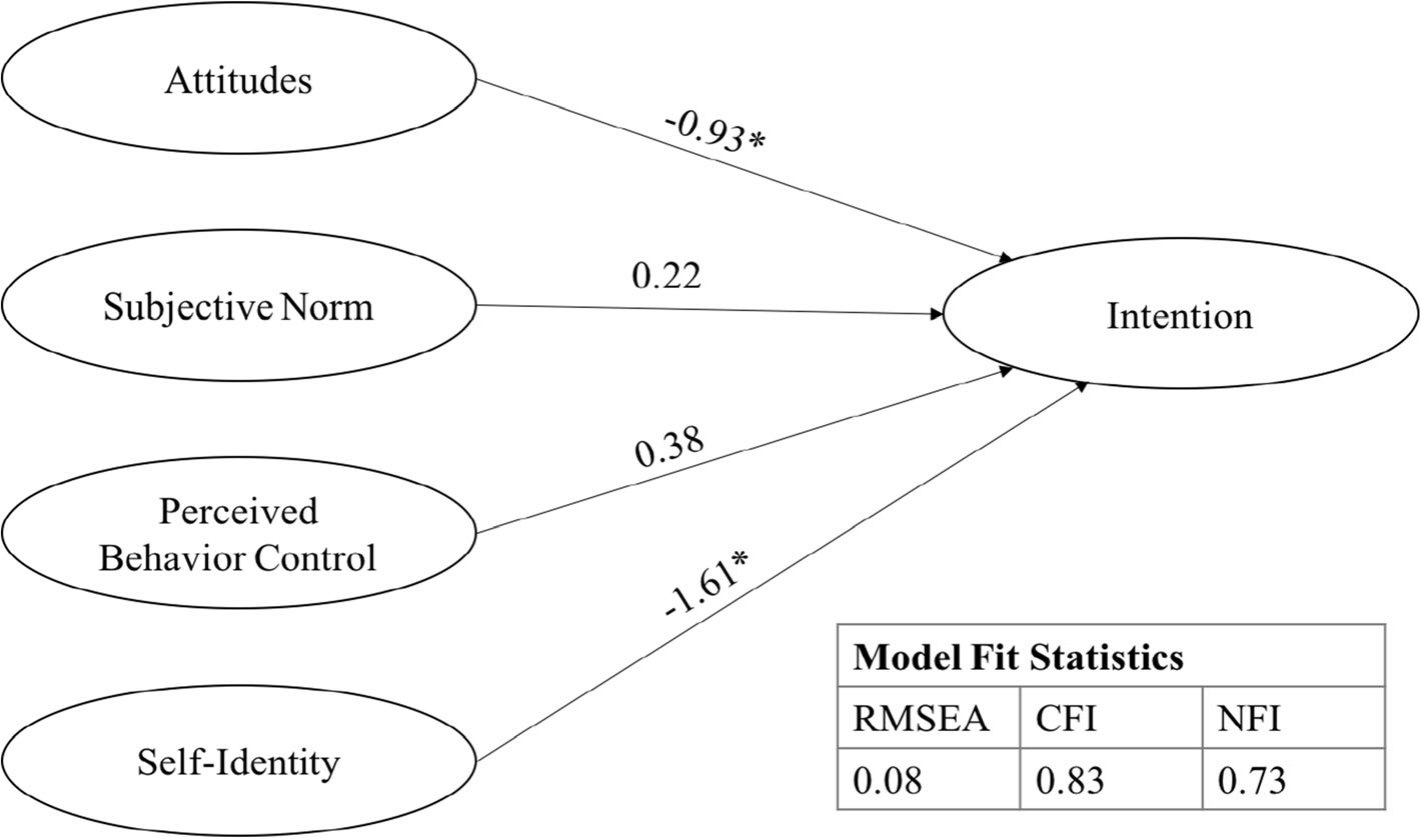
TPB model with self-identity extension. SEM results with latent factor of self-identity. Note: Independent, latent variable covariance paths not shown for simplicity

The self-identity construct and its relationship with intention to switch to a LARC method (Fig. 2) was then tested as an extension of the TPB. Including self-identity improved model fit and provided further support for the TPB model. Model Fit Statistics indicated χ2 (866, *N* = 186) *p* < 0.01, CFI of 0.83, and NFI of 0.73. The model produced an RMSEA of 0.08 with a 90% confidence interval of 0.08 to 0.09. Attitude and self-identity were significant (*p* < 0.01) in influencing LARC intention at − 0.9 and − 1.6, respectively. Path coefficients for perceived behavioral control and subjective norm decreased to 0.4 and 0.2, with no significance, which is consistent with previous LARC research [8]. All intention items were significant in measuring the factor, indicating LARC intention was accurately measured (Table 2).

**Table 2:** Variable item estimates. Variable estimates and their significance

Independent Variables								Dependent Variable	
Attitude	Est.	Subjective Norm	Est.	Perceived Behavioral Control	Est.	Self-Identity	Est.	Intention	Est.
A1	0.23*	SN1	0.85**	PC1	−0.64	SI1	0.11	I1	0.81**
A2	0.32*	SN2	0.86**	PC2	−0.86	SI2	−0.80**	I2	0.71**
A3	0.191*	SN3	0.63**	PC3	−0.90	SI3	0.49**	I3	0.72**
A4	0.820*	SN4	0.87**	PC4	−0.72	SI4	0.24*	I4	0.77**
A5	0.685*	SN5	0.60**	PC5	−0.48	SI5	0.48	I5	0.88**
A6	0.75*			PC6	−0.44			I6	0.88**
A7	0.77*							I7	0.77**
A8	0.69*							I8	0.62**
A9	0.61*							I9	0.70**
A10	0.46*							I10	0.75**
A11	0.50*							I11	0.81**
A12	0.38*							I12	0.88**
								I13	0.89**
								I14	0.88**
								I15	−0.73**
								I16	−0.75**

Note: Items listed as TPB construct and corresponding number associated with survey item

*significant at *p* < 0.05

**significant at *p* < 0.01

### Multicollinearity

High regression weights may be associated with TPB factors’ multicollinearity, and can generate negative covariance when using SEM [33]. Table 3 outlines estimate paths of all factors. Attitude and subjective norm (0.8, *p* < 0.05), and attitude and self-identity (− 0.9, *p* < 0.05), were correlated in the final TPB extended model. Subjective norm and self-identity paths remained negative; therefore, conclusions drawn from the extended TPB model are valid. Pearson correlation coefficients for some items were correlated (Table 4). A collinearity diagnostic test (Table 5), however, revealed Variable Inflation Indices (VIF) less than 10, and variance proportions of all factors were less than 0.9, indicating the model does not violate the SEM assumption [33]. Similar results occurred when the collinearity diagnostic test ran with individual item measures.

**Table 3:** Factor covariance and correlations. Factor covariance and correlations

Factor Paths	Covariance		Correlations
	Est.	S.E	Est.
Attitude<−- > Subjective Norm	0.33*	0.12	0.79
Subjective Norm <−- > Perceived Behavioral Control	−0.02	0.06	−0.39
Attitude <−- > Perceived Behavioral Control	− 0.01	0.01	− 0.24
Self-Identity <−- > Perceived Behavioral Control	0.01	0.02	0.13
Self-Identity <−- > Subjective Norm	−0.56**	0.13	−0.67
Self-Identity <−- > Attitude	−0.29*	0.11	−0.89

*significant at *p* < 0.05

**significant at *p* < 0.01

**Table 4:** Pearson correlation coefficients among TPB constructs

Attitude	A1	A2	A3	A4	A5	A6	A7	A8	A9	A10	A11	A12
A1	1											
A2	0.27^**^	1										
A3	0.16^*^	0.62^**^	1									
A4	0.15^*^	0.23^**^	0.12	1								
A5	0.28^**^	0.25^**^	0.12	0.61^**^	1							
A6	0.19^**^	0.16^*^	0.04	0.72^**^	0.56^**^	1						
A7	0.20^**^	0.20^**^	0.13	0.76^**^	0.54^**^	0.79^**^	1					
A8	0.15^*^	0.24^**^	0.16^*^	0.55^**^	0.49^**^	0.45^**^	0.45^**^	1				
A9	0.03	0.17^*^	0.08	0.50^**^	0.32^**^	0.38^**^	0.39^**^	0.50^**^	1			
A10	0.09	0.12	0.07	0.35^**^	0.20^**^	0.25^**^	0.33^**^	0.30^**^	0.44^**^	1		
A11	0.14	0.23^**^	0.20^**^	0.41^**^	0.31^**^	0.28^**^	0.40^**^	0.27^**^	0.35^**^	0.67^**^	1	
A12	0.05	0.19^**^	0.18^*^	0.24^**^	0.19^*^	0.20^**^	0.25^**^	0.17^*^	0.29^**^	0.42^**^	0.55^**^	1
Subjective Norm	SN1	SN2	SN3	SN4	SN5							
SN1	1											
SN2	0.69^**^	1										
SN3	0.48^**^	0.54^**^	1									
SN4	0.74^**^	0.77^**^	0.60^**^	1								
SN5	0.47^**^	0.54^**^	0.33^**^	0.52^**^	1							
Perceived Behavioral Control	PC1	PC2	PC3	PC4	PC5	PC6						
PC1	1											
PC2	0.51^**^	1										
PC3	0.54^**^	0.82^**^	1									
PC4	0.50^**^	0.54^**^	0.50^**^	1								
PC5	0.46^**^	0.35^**^	0.40^**^	0.44^**^	1							
PC6	0.49^**^	0.37^**^	0.33^**^	0.39^**^	0.33^**^	1						
Self-Identity	SI1	SI2	SI3	SI4	SI5							
SI1	1											
SI2	−0.13	1										
SI3	0.28^**^	−0.53^**^	1									
SI4	0.19^*^	−0.29^**^	0.33^**^	1								
SI5	0.26^**^	−0.41^**^	0.45^**^	0.36^**^	1							

*Correlation is significant at the *p* < 0.05 level (2-tailed)

**Correlation is significant at the *p* < 0.01 level (2-tailed)

**Table 5:** Multicollinearity diagnostic test, variance proportions

Dim.	(Constant)	Attitude	Subjective Norm	Perceived Behavioral Control	Self-Identity
VIF		2.37	2.20	1.27	1.20
1	0.00	0.00	0.00	0.00	0.00
2	0.00	0.02	0.06	0.00	0.32
3	0.00	0.02	0.23	0.82	0.10
4	0.22	0.31	0.56	0.15	0.15
5	0.78	0.65	0.15	0.02	0.42

## Discussion

The current study used SEM to examine the relationship of COC users’ self-identity as an extension of the TPB to predict LARC intention. The TPB model alone and the self-identity extension provided acceptable model fit statistics. Results indicate participants’ attitudes, behaviors, perceived behavioral control, and self-identity influence LARC intention. Findings provide a deeper understanding of the paradox of inertia and can be used to address resistance to change.

Our study revealed COC users were more likely to ask LARC users about their experiences than discussing it with their physician, sexual partner, mother, or friend. This confirms developing LARC messaging highlighting user experience to influence uptake can overcome the paradox of inertia (e.g., providing quotes from experienced LARC users); supporting previous findings on LARC campaign effectiveness [13]. COC users indicated sexual partners would support LARC use more than their mothers or friends, suggesting partners may influence contraceptive choice and could represent an important secondary audience in LARC health education interventions. Participants’ intention to discuss LARC method adoption with physicians was significant, demonstrating the weight of physician support, recommendation, and knowledge on women’s contraceptive choices. Additional LARC interventions should focus on improving physician’s access to accurate and updated information regarding LARC methods, addressing the barrier of physicians’ perceived lack of knowledge and confidence in LARC recommendations [4, 14, 15].

Attitude had a significant, negative relationship with LARC intention when self-identity was added to the TPB model. Reinforcing the paradox of inertia, COC users’ positive attitude toward LARC had a negative effect on overall intention— inconsistent with previous studies [8]. This may be due to previous studies’ inclusion of all prescription contraceptive users (including LARC methods) and their attitudes after viewing LARC messaging campaigns. This demonstrates the impact of access to contraceptive choice information, and the strength of self-identity among COC users without exposure to LARC messages. COC user’s positive attitudes influenced intentions to inquire about LARC, though overall LARC intent was lowered when other factors were considered. This emphasizes those with greater attitudes are more likely to seek information [34], and are more likely to resist change without this information; highlighting the significance of improving remaining barriers to LARC information [8, 34]. Future research should employ SEM to explore whether similar relationships occur after exposing COC users to LARC messaging campaigns. A deeper understanding of the strength of COC identity and the ways in which the paradox of inertia can be alleviated may be provided; moving beyond improving attitudes toward LARC methods.

Self-identity with being a pill user also had a significant, negative relationship with LARC intention when adding the variable to the TPB model, as expected. As the association between COC use and a COC user’s self-identity increased, overall LARC intentions decreased. Therefore, how a woman identifies with being a pill user (i.e., someone who likes the responsibility of taking a pill every day, prefers menses control, views the pill as part of their female identity) may influence her willingness to switch to an alternative contraceptive method, such as LARC. Prior paradox of inertia research has found the invasive nature of LARC insertion is an uptake barrier [15]. LARC insertion may lead to a sense of permanence (thus removing the daily contraceptive engagement and menses control) and may be too drastic of a change to a woman’s self-concept. The perceived permanence may be equivalent to an unchanging/objective characteristic (i.e., nationality, gender, and age) [35], where COCs have the ability to evolve with an individual and their social behaviors. As motivations to use contraceptives are driven by evolving social factors (e.g., community norms, sexual relationship status); the paradox lies within— not exclusively the resistance to change— but a resistance to permanence. LARC insertion may be viewed with a permanence equivalent to other procedures related to a woman’s self-identity (e.g., hysterectomy) [36], although these relationships have not been significantly proven. Resistance to permanence may explain why including self-identity was influential as an extension of the TPB and overall LARC intention.

A COC user may relate COC’s habitual and controllable consumption to other health products (e.g., menstrual hygiene management products) used as part of their personal space or bodies. Products that are self-perceived as an extension of an individual (i.e., their extended self) [37, 38], are often associated with a sense of loss when eliminated. COC users, who may associate taking the pill everyday as an extension of themselves or as evidence of personality traits (e.g., being responsible [32]), may feel their identity is violated when this behavior is eliminated, as they are no longer able to have significant control over their cycle. A sense of risk may be further exacerbated by LARC’s potential to limit monthly menstruation [39], an important aspect of a woman’s self-concept as these findings suggest. Accurate LARC information may provide closure, reduce confusion and/or uncertainty, and lessen the sense of loss, similar to what is experienced with other constructs related to the extended self (e.g., sexual history, cigarette use, pet ownership, having children) [40]. Future research on women’s perceptions of LARC methods in relation to resistance to other reproductive health procedures resulting in permanence and loss of self (e.g., hysterectomy, tubal ligation, etc.) should be explored.

Current COC user’s intention to switch to a LARC method in the prolonged future proved greater than intention for immediate use (12 months). Further explaining the paradox of inertia, a change to a habitual health behavior may come over time when the practice is related to a woman’s self-concept and evolving social influencers. Although COC users are aware of LARC, initial resistance may be evident. With exposure to accurate information from experienced LARC users, support of sexual partners, and effective contraceptive counseling, this resistance may decrease over time.

## Conclusions

A woman’s self-identity can influence contraceptive choice, indicating the importance of personalized contraceptive counseling approach adoption. Though this study captured insights specific to the southeastern United States female undergraduate student target demographic, geographic and demographic limitations do exist. Given the homogeneity of the study population, generalizability across the United States is limited. Recruitment tactics also pose further limitations, as convenience sampling and self-selection bring about biases that may impact study results. While these limitations may be viewed as detriments to the current study, the results nonetheless provide direction for health professionals and practitioners surrounding LARC education and counseling for college-aged women. The current research may also inform future LARC communication campaigns and interventions by providing targeted and effective messaging content recommendations. Specifically, campaigns should include LARC users’ positive experiences (in their own words) and focus on LARC benefits (such as responsibility) to prevent unintended pregnancies. Messaging should also include how LARC is an important part of who a woman is and her control over body. Applications of these findings may help daily contraceptive method (e.g., COC) users overcome the paradox of inertia in switching to a non-daily method (e.g., LARC), and providing one opportunity to decrease unintended pregnancy rates in the United States.

## Additional file

Additional file 1: Web-based survey via Qualtrics. (PDF 1412 kb)

## Data Availability

The datasets used and analyzed during the current study are available from the corresponding author on reasonable request.

## Abbreviations

COC: Combined oral contraceptive pill
FDA: Food and Drug Administration
IUD: Intrauterine device
LARC: Long-acting reversible contraceptive
SEM: Structural equation modeling
TPB: Theory of Planned Behavior
