# Supplementary material for: Using the theory of planned behavior and self-identity to explore women’s decision-making and intention to switch from combined oral contraceptive pill (COC) to long-acting reversible contraceptive (LARC)
Source: BMC Womens Health. 2019 Jun 20;19:82. doi: 10.1186/s12905-019-0772-8 (PMC6585137; doi:10.1186/s12905-019-0772-8)
Supplement: Supplementary file 1 — Web-based survey via Qualtrics. (PDF 1412 kb) [file 12905_2019_772_MOESM1_ESM.pdf]

Default Question Block

You are invited to participate in a research study. This research, conducted by Beth L. Sundstrom, Assistant Professor, Communication, Grace E. Moxley, and the Women's Health Research Team of the College of Charleston, is designed to improve understandings of knowledge, attitudes, and behaviors related to contraceptive methods, including the IUD and Implant. Current female undergraduate students of the College of Charleston are invited to participate in this study.

Participation in this study will require about 15-20 minutes of your time. As a participant in this research, you will be asked to complete a web-based survey.

Your responses will be anonymous. We will keep all information strictly confidential and destroy it when the research is complete. At no time will you be able to be identified in any reports or publications resulting from this research.

Although it is not anticipated that you will benefit directly through your involvement in this study, this research is expected to benefit women through better understanding of contraception, which may allow health care providers to better serve individual women.

There is minimal risk associated with this study. Should you experience any psychological or social distress from your participation, please contact College of Charleston Health Services at healthservices@cofc.edu, or your preferred medical provider.

Your participation is completely voluntary, and you may discontinue participation at any time.

Upon completion of the survey, you will be able to choose to be entered into a drawing to win a gift basket from Lush valued at around $30. Your contact information will be collected in a separate survey and will remain unidentified with your responses to this survey.

If you have any questions concerning this research study, please contact Beth Sundstrom at sundstrombl@cofc.edu. You may also contact Research Protections & Compliance on the Office of Research and Grants Administration, at 843-953-7421 or e-mail compliance@cofc.edu if you have questions or concerns about research review at the College of Charleston or your rights as a research participant.

This research has been approved by the College of Charleston Institutional Review Board for the Protection of the Human Research Participants. IRB Approval Code: **GMGM-10-24-2016**

I have read this consent form, and by clicking the arrows below, I agree to participate in this research study and certify that I am at least 18 years old.

Are you currently a female undergraduate student at the College of Charleston?

Yes

No

If you are currently using a contraceptive method, what would you say is your primary method?

Birth Control Pill

Cervical Cap

Condom

Diaphragm

Emergency Contraception (Plan B®)

Female Sterilization (Tying tubes, Essure®)

Implant (Implanon®, Nexplanon®)

IUD (Mirena®, ParaGard®, Skyla®)

Male Sterilization (Vasectomy)

Natural Family Planning Method or Rhythm Method

Patch (Ortho Evra)

Shot (Depo-Provera or Lunelle)

Spermicide

Sponge

Vaginal Ring (Nuva Ring®)

Withdrawal Method (Pull out Method)

[ ] Other

I am not currently using a contraceptive method

How long has the pill been your primary contraceptive method?

Years [ ]

Months [ ]

Have you ever heard of the contraceptive method called the IUD (intrauterine device; e.g. Mirena, Paraguard, Liletta, Skyla)?

Yes

No

Have you ever heard of the contraceptive method called the Implant (e.g., Nexplanon, Implanon)?

Yes

No

To me, obtaining an IUD or Implant seems

	1	2	3	4	5	6	7	
Extremely Difficult	○	○	○	○	○	○	○	Extremely Easy
Extremely Frightening	○	○	○	○	○	○	○	Extremely Comforting
Extremely Painful	○	○	○	○	○	○	○	Extremely Painless

Choosing an IUD or Implant as my primary birth control method would be

	1	2	3	4	5	6	7	
Extremely Harmful	○	○	○	○	○	○	○	Extremely Beneficial
Extremely Inconvenient	○	○	○	○	○	○	○	Extremely Convenient
Extremely Irresponsible	○	○	○	○	○	○	○	Extremely Responsible
Extremely Unhealthy	○	○	○	○	○	○	○	Extremely Healthy

Using an IUD or Implant would be more convenient for my lifestyle than other contraceptive methods, such as the pill.

	1	2	3	4	5	6	7	
Strongly Disagree	○	○	○	○	○	○	○	Strongly Agree

IUDs and Implants are more effective at preventing pregnancy than other contraceptive methods, such as the pill.

	1	2	3	4	5	6	7	
Strongly Disagree	○	○	○	○	○	○	○	Strongly Agree

An IUD or Implant would be able to control my acne as well as other contraceptive methods, such as the pill.

	1	2	3	4	5	6	7	
Strongly Disagree	○	○	○	○	○	○	○	Strongly Agree

An IUD or Implant would be as effective at reducing menstrual cramps as other contraceptive methods, such as the pill.

	1	2	3	4	5	6	7	
Strongly Disagree	○	○	○	○	○	○	○	Strongly Agree

An IUD or Implant would be as effective at regulating my menstrual cycle as other contraceptive methods, such as the pill.

	1	2	3	4	5	6	7	
Strongly Disagree	○	○	○	○	○	○	○	Strongly Agree

The IUD and Implant are good contraceptive options for women in my peer group.

	1	2	3	4	5	6	7	
Strongly Disagree	○	○	○	○	○	○	○	Strongly Agree

My friends would support my decision to use an IUD or Implant as my primary contraceptive method.

	1	2	3	4	5	6	7	
Extremely Unlikely	○	○	○	○	○	○	○	Extremely Likely

My mother or female mentor would support my decision to use an IUD or Implant as my primary contraceptive method.

	1	2	3	4	5	6	7	
Extremely Unlikely	○	○	○	○	○	○	○	Extremely Likely

My sexual partner would support my decision to use an IUD or Implant as my primary contraceptive method.

If you are not currently sexually active, please leave this question blank.

1 2 3 4 5 6 7

Extremely Unlikely	○	○	○	○	○	○	○	○	Extremely Likely

If I used an IUD or Implant as my primary contraceptive method, I would be able to relate to my peers.

	1	2	3	4	5	6	7	
Strongly Disagree	○	○	○	○	○	○	○	Strongly Agree

I am confident in my ability to obtain an IUD or Implant if I desired to use it as my primary contraceptive method.

	1	2	3	4	5	6	7	
Strongly Disagree	○	○	○	○	○	○	○	Strongly Agree

Obtaining an IUD or Implant as my primary contraceptive method would be:

	1	2	3	4	5	6	7	
Not at all up to me	○	○	○	○	○	○	○	Completely up to me
Not at all under my control	○	○	○	○	○	○	○	Completely under my control

My health care provider would support my decision to obtain an IUD or Implant.

If you do not have a health care provider, please leave this question blank.

	1	2	3	4	5	6	7	
Extremely Unlikely	○	○	○	○	○	○	○	Extremely Likely

My health insurance would pay for an IUD or Implant.

If you do not have health insurance, please leave this question blank.

	1	2	3	4	5	6	7	
Extremely Unlikely	○	○	○	○	○	○	○	Extremely Likely

I know where to go if I wished to obtain an IUD or Implant as my primary contraceptive method.

	1	2	3	4	5	6	7	
Strongly Disagree	○	○	○	○	○	○	○	Strongly Agree

Taking the pill every day is an important part of who I am.

	1	2	3	4	5	6	7	
Strongly Disagree	○	○	○	○	○	○	○	Strongly Agree

I am the type of woman who would use an IUD or Implant as my primary birth control method.

	1	2	3	4	5	6	7	
Strongly Disagree	○	○	○	○	○	○	○	Strongly Agree

I see myself as responsible enough to take the pill every day; the IUD and Implant are not for women like me.

	1	2	3	4	5	6	7	
Strongly Disagree	○	○	○	○	○	○	○	Strongly Agree

Having a monthly period is an important part of my identity as a woman.

	1	2	3	4	5	6	7	
Strongly Disagree	○	○	○	○	○	○	○	Strongly Agree

Taking the pill every day protects my future fertility better than other contraceptive methods, like the IUD or Implant.

	1	2	3	4	5	6	7	
Strongly Disagree	○	○	○	○	○	○	○	Strongly Agree

I intend to research more about the IUD or Implant in the future.

	1	2	3	4	5	6	7	
Strongly Disagree	○	○	○	○	○	○	○	Strongly Agree

I intend to research if my insurance plan covers the IUD or Implant.

	1	2	3	4	5	6	7	
Strongly Disagree	○	○	○	○	○	○	○	Strongly Agree

I intend to research facilities in my area that offer IUD or Implant insertions.

	1	2	3	4	5	6	7	
Strongly Disagree	○	○	○	○	○	○	○	Strongly Agree

I intend to discuss the IUD or Implant as an option for my primary form of contraception with my health care provider at my next appointment.

	1	2	3	4	5	6	7	
Strongly Disagree	○	○	○	○	○	○	○	Strongly Agree

I intend to discuss the IUD or Implant as an option for my primary form of contraception with my health care provider in the future.

	1	2	3	4	5	6	7	
Strongly Disagree	○	○	○	○	○	○	○	Strongly Agree

I intend to learn more about the process of getting an IUD or Implant and how I may go about obtaining one in the future.

	1	2	3	4	5	6	7	
Strongly Disagree	○	○	○	○	○	○	○	Strongly Agree

I intend to talk to my friends about the IUD or Implant in the future.

	1	2	3	4	5	6	7	
Strongly Disagree	○	○	○	○	○	○	○	Strongly Agree

I intend to talk to my mom or female mentor about the IUD or Implant in the future.

	1	2	3	4	5	6	7	
Strongly Disagree	○	○	○	○	○	○	○	Strongly Agree

I intend to talk to my partner about the IUD or Implant in the future.

	1	2	3	4	5	6	7	
Strongly Disagree	○	○	○	○	○	○	○	Strongly Agree

I intend to ask IUD and Implant users about their experiences with the methods to determine if an IUD or Implant might be right for me.

	1	2	3	4	5	6	7	
Strongly Disagree	○	○	○	○	○	○	○	Strongly Agree

I can see myself using an IUD or Implant as my primary contraceptive method in the next 12 months.

	1	2	3	4	5	6	7	
Strongly Disagree	○	○	○	○	○	○	○	Strongly Agree

I can see myself using an IUD or Implant as my primary contraceptive method in the future.

	1	2	3	4	5	6	7	
Strongly Disagree	○	○	○	○	○	○	○	Strongly Agree

I intend to take the steps to obtain an IUD or Implant as my primary contraceptive method in the future.

	1	2	3	4	5	6	7	
Strongly Disagree	○	○	○	○	○	○	○	Strongly Agree

I intend to change my contraceptive method from the pill to the IUD or Implant.

	1	2	3	4	5	6	7	
Strongly Disagree	○	○	○	○	○	○	○	Strongly Agree

I am happy with the pill, and I do not intend to change my method in the future.

	1	2	3	4	5	6	7	
Strongly Disagree	○	○	○	○	○	○	○	Strongly Agree

I have chosen the pill as my primary contraceptive method, and I do not intend to change.

	1	2	3	4	5	6	7	
Strongly Disagree	○	○	○	○	○	○	○	Strongly Agree

I generally consider changes to be a negative thing.

	1	2	3	4	5	6	7	
Strongly Disagree	○	○	○	○	○	○	○	Strongly Agree

I'll take a routine day over a day full of unexpected events any time.

	1	2	3	4	5	6	7	
Strongly Disagree	○	○	○	○	○	○	○	Strongly Agree

I like to do the same old things rather than try new and different ones.

	1	2	3	4	5	6	7	
Strongly Disagree	○	○	○	○	○	○	○	Strongly Agree

I'd rather be bored than surprised.

	1	2	3	4	5	6	7	
Strongly Disagree	○	○	○	○	○	○	○	Strongly Agree

Whenever my life forms a stable routine, I look for ways to change it.

	1	2	3	4	5	6	7	
Strongly Disagree	○	○	○	○	○	○	○	Strongly Agree

If I were to be informed that there's going to be a significant change regarding the way things are done at work or school, I would probably feel stressed.

	1	2	3	4	5	6	7	
Strongly Disagree	○	○	○	○	○	○	○	Strongly Agree

When I am informed of a change of plans, I tense up a bit.

	1	2	3	4	5	6	7	
Strongly Disagree	○	○	○	○	○	○	○	Strongly Agree

When things don't go according to plans, it stresses me out.

	1	2	3	4	5	6	7	
Strongly Disagree	○	○	○	○	○	○	○	Strongly Agree

If my boss/professor changed the criteria for evaluating employees/students, it would probably make me feel uncomfortable even if I thought I'd do just as well without having to do any extra work.

	1	2	3	4	5	6	7	
Strongly Disagree	○	○	○	○	○	○	○	Strongly Agree

Changing plans seems like a real hassle to me.

	1	2	3	4	5	6	7	
Strongly Disagree	○	○	○	○	○	○	○	Strongly Agree

Often, I feel a bit uncomfortable even about changes that may potentially improve my life.

	1	2	3	4	5	6	7	
Strongly Disagree	○	○	○	○	○	○	○	Strongly Agree

When someone pressures me to change something, I tend to resist it even if I think the change may ultimately benefit me.

	1	2	3	4	5	6	7	
Strongly Disagree	○	○	○	○	○	○	○	Strongly Agree

I sometimes find myself avoiding changes that I know will be good for me.

	1	2	3	4	5	6	7	
Strongly Disagree	○	○	○	○	○	○	○	Strongly Agree

I often change my mind.

	1	2	3	4	5	6	7	
Strongly Disagree	○	○	○	○	○	○	○	Strongly Agree

Once I've come to a conclusion, I'm not likely to change my mind.

	1	2	3	4	5	6	7	
Strongly Disagree	○	○	○	○	○	○	○	Strongly Agree

I don't change my mind easily.

	1	2	3	4	5	6	7	
Strongly Disagree	○	○	○	○	○	○	○	Strongly Agree

My views are very consistent over time.

	1	2	3	4	5	6	7	
Strongly Disagree	○	○	○	○	○	○	○	Strongly Agree

How old are you? Please type your age in years (e.g., 18)

What is your classification?

Freshman

Sophomore

Junior

Senior

Which of the following best describes your race/ethnicity? Please mark all that apply.

American Indian or Alaskan Native

Asian or Asian American

Black or African American

Native Hawaiian or Other Pacific Islander

Hispanic or Latino

White or Caucasian

Other

Which of the following commonly used terms best describes your sexual orientation?

Heterosexual/Straight

Homosexual/Gay or Lesbian

Bisexual

Asexual (I have never been sexually attracted to others)

Intersex

Queer

[ ] Other

Are you currently covered by any form of health insurance or health plan? If you are not currently covered by any form of insurance, please leave the question blank.

Private insurance

Medicaid, the government program that helps pay medical bills for people with low incomes

Some other government medical program, such as Medicare, CHAMPUS, or the VA

[ ] Other

Do you have an OB/GYN who you see regularly for gynecological care?

Yes

No

Have you ever had surgery (e.g. tonsillectomy, appendectomy, wisdom teeth removal, etc.)?

Yes

No

Have you ever been admitted to the hospital and had to stay for a few hours or more?

Yes

No

Have you ever had <u>vaginal intercourse</u> (penis inserted into your vagina)?

Yes

No

During the <u>past year</u>, how many <u>vaginal intercourse</u> partners did you have?

[ ]

Have you ever been pregnant? If yes, please indicate the number of times you have been pregnant.

[ ] Yes

No

How many of your pregnancies were unplanned?

[ ] Number of unplanned pregnancies

I did not have any unplanned pregnancies

How many times, if ever, have you experienced the following? Please indicate the number below each response option.

Ectopic Pregnancy

Miscarriage

Abortion

I have not experienced an ectopic pregnancy, miscarriage, or abortion

Thank you for completing the Women's Health Research Team survey!

You can find out more about our research team by visiting hss.cofc.edu/whrt.

Would you like to provide your name, phone number, and email for the chance to win a gift basket from Lush valued at around $30? If you select yes, you will be taken to a second survey so that you can enter your contact information. Your contact information will remain unidentified with your responses to this survey.

Yes

No

We thank you for your time spent taking the Women's Health Research Team Survey.

Should you have further questions about the study, please contact Beth Sundstrom (sundstrombl@cofc.edu).

Please click the arrows below so that your response can be recorded.

Powered by Qualtrics
